# An English Flipped Classroom Teaching Model Based on Big Data Analysis

**DOI:** 10.1155/2022/7391258

**Published:** 2022-08-31

**Authors:** Wang Sa, Li Dan, Du Juan, Yu Fei, Wang Wei

**Affiliations:** ^1^School of Foreign Language, Beihua University, Jilin 132011, China; ^2^School of Literature and Journalism, Yantai University, Yantai, Shangdong 264005, China

## Abstract

In order to improve the defect that the quality of English flipped classroom teaching cannot be quantitatively evaluated, an English flipped classroom teaching model based on big data learning analysis is proposed. In the English flipped classroom teaching mode, which applies the flipped classroom teaching mode, the classroom teaching links are changed, the preview feedback, joint answer and question between teachers and students, classroom teaching, and teachers' questions are taken as the key links of classroom teaching, and the teacher education and school management system are improved, so as to complete the reform of English flipped classroom teaching mode. The convolution neural network is used to extract the evaluation text features, mine the association rules of massive evaluation text data through the Apriori algorithm, determine the evaluation index of English flipped classroom teaching quality, and complete the evaluation of English flipped classroom teaching quality by using the decision tree method in big data analysis. The experimental results show that the proposed method can quantitatively evaluate the quality of English flipped classroom teaching by using the evaluation text, and the evaluation accuracy and recall rate are higher than 98%, which can realize the objective evaluation of English flipped classroom teaching quality.

## 1. Introduction

At present, many international conferences are communicated in English [1], and many materials are published in English. Even many letters and even broadcasts are inseparable from English. It can be seen that English is widely used [2, 3]. Therefore, in the new era of social development, English has gradually become the focus of attention and one of the important indicators of talent training in colleges and universities. In the information age, with the rapid development of information technology, on the one hand, it has brought new opportunities for college English teaching. On the other hand, it also makes college English teaching face new challenges. Therefore, it is a problem faced by major colleges and universities to establish an English flipped classroom teaching model in line with the development of the times under the background of informatization [4, 5].

Reference [6] uses the comparative method to analyze the two types of assessment: formative assessment and summative assessment. The results show that the characteristics of formative evaluation include teachers' adaptability to the classroom and immediate feedback to teachers. As a high-risk evaluation, summative evaluation needs high standard control and safety to ensure its reliability and effectiveness. In [7], through one-on-one cooperation with speech and language therapy students, children participated in weekly activities aiming at promoting receptive and productive knowledge of 20 target words in secondary vocabulary categories. Reference [8] adopts an online format and carries out digital access through the school-oriented learning management system. Upon completion, teachers will receive a personalized report on their ability to assess teaching skills in nonuniversity teaching.

Considering that, at present, the evaluation of English flipped classroom teaching quality is mainly achieved through a small amount of data, and the evaluation results under the condition of small sample data have high limitations and one sidedness. Big data technology can effectively improve this defect. Big data technology has transformed from sampling evaluation of English flipped classroom teaching quality to full data evaluation of English flipped classroom teaching quality. Mining teaching quality related information from massive data to improve the diversity of English classroom teaching quality evaluation [9]. Therefore, on the basis of clarifying the English flipped classroom teaching mode, this paper uses big data technology to evaluate the teaching quality of English flipped classroom.

## 2. Reform of English Flipped Classroom Teaching Mode

### 2.1. Changing Classroom Teaching Links

**Figure 1:**
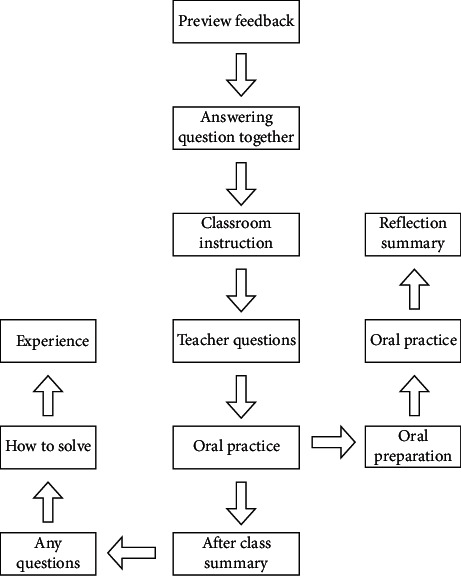
Process design of flipped classroom teaching.

Under the traditional teaching mode, the low efficiency of traditional classroom teaching restricts the improvement of classroom teaching quality. At the same time, some students have relatively weak basic knowledge, weak learning ability, and low learning enthusiasm [10, 11]. In order to effectively improve classroom efficiency and improve students' knowledge level, it is urgent to reform classroom teaching. Therefore, we should change the traditional classroom teaching links. It is transformed into a teaching link of preview feedback, joint answer and question between teachers and students, classroom teaching, teachers' questions, students' oral training, classroom summary, and reflection, as shown in Figure 1.

**Figure 2:**
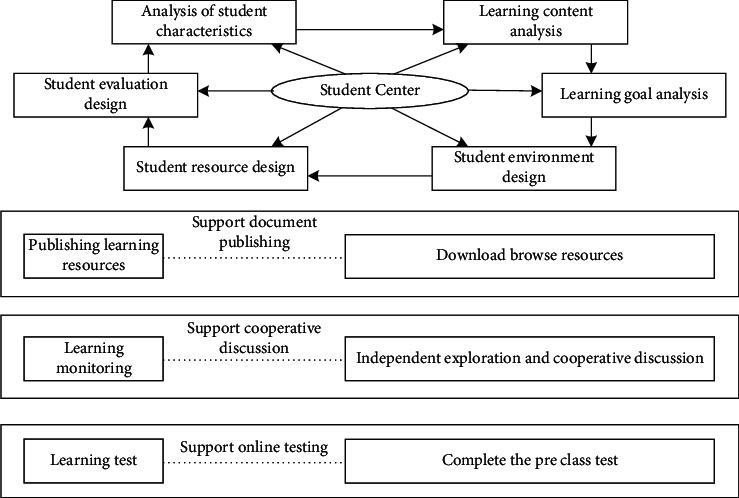
Design of learning activities in preview feedback stage.

Through preview feedback, teachers can quickly grasp the learning situation of students and help teachers explain in class. The main contents contained in preview feedback are shown in Figure 2.

First, the teacher organizes the students to have a group discussion [12], the team leader writes down the questions, and then the team leader asks the teachers. Through the above methods, the teachers can understand the students' learning situation and can also use these questions as a reference for subsequent classroom questioning and teaching. In the preview process, it is difficult for students to grasp the overall knowledge and logic, and it is inevitable that they will not fully understand the knowledge points and key points. Therefore, teachers need to check the lack of formulas, ask questions to students, make students' thinking active, and stimulate students' learning enthusiasm at the same time.

### 2.2. Improve Teacher Education and School Management System

The traditional teaching mode is that teachers teach according to textbooks. In the information age, teachers' teaching ability needs to be improved. At the same time, the traditional school management system is mainly managed by the school. Under the flipped classroom teaching mode [13], it not only puts forward higher requirements for teachers' ability but also puts forward higher requirements for school management.

**Figure 3:**
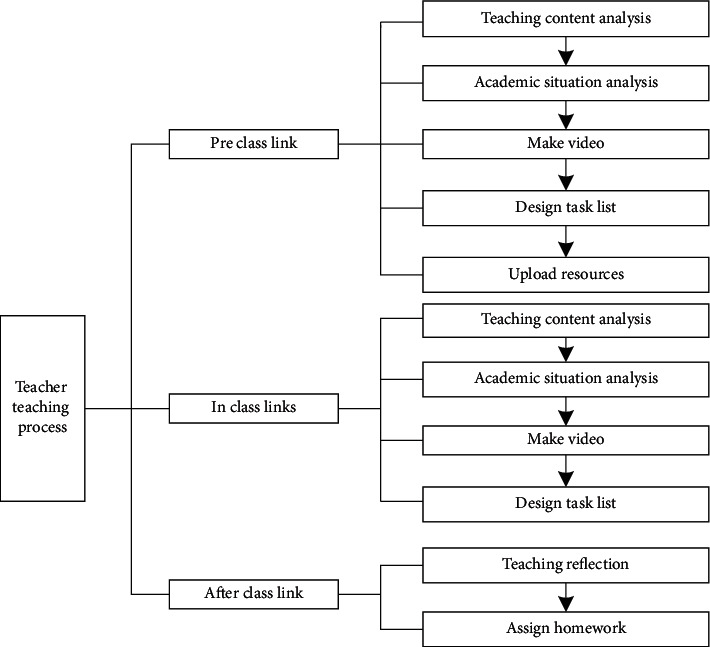
Teacher teaching process.

In terms of improving the teacher education system, teachers are required to turn from the person who prepares the teaching plan to the video producer, from the lecturer on the podium to the communicator among students, and from the outsider who internalizes students' knowledge to the guide in the classroom. The main teaching process of teachers in class is shown in Figure 3.

At the same time, the flipped classroom teaching mode pushes teachers and students to the front end of the network. Only by improving their ability in the information age can teachers better understand students and meet students' learning needs in the network environment [14, 15]. Through the above analysis, according to the requirements of English teaching, in order to improve the problem that the quality of English flipped classroom teaching cannot be quantitatively evaluated, the evaluation criteria of teachers' learning tasks are formulated according to Table 1.

Based on the analysis of the current school management system, parents are more dependent on the school management system. Because their right to participate in school management is limited, they will not take the initiative to fulfill their obligations to students' learning. The emergence of flipped classroom teaching mode divides the traditional teaching process into two parts, in class and after class [16, 17]; that is, the right to guide students' learning is pushed to parents. In order to better promote flipped classroom in China, innovate at the level of the school management system, tap the role of parents in the process of education and teaching, enable parents to give full play to their talents in the process of school running, and jointly promote the growth of students, so as to promote the application of flipped classroom teaching mode from the level of school management system.

## 3. Evaluation of English Flipped Classroom Teaching Quality Based on Big Data Technology

### 3.1. Evaluation Text Feature Extraction

The convolution neural network method is selected to extract the text features of English flipped classroom teaching quality evaluation. The convolution neural network automatically learns the input data features through convolution layer and pool layer. The training set is established by using the evaluation text, and all samples in the training set are marked by tags. After inputting the test dataset, the trained neural network is used to obtain the classification label of new samples [18]. Convolution neural network method not only has the characteristics of analyzing the context semantics of comment text and feature learning but also has the advantages of high noise resistance and high classification degree.

**Figure 4:**
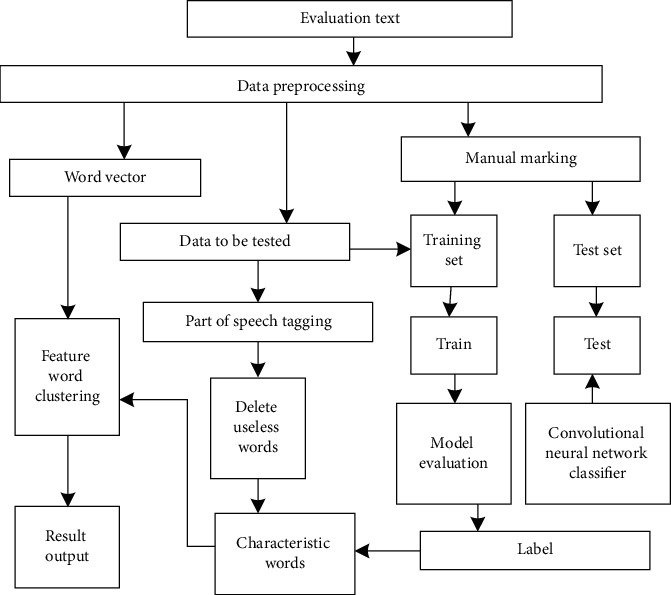
Structure diagram of evaluation text feature extraction.

The feature structure of the evaluation text is extracted by convolution neural network, as shown in Figure 4.

As can be seen from Figure 4, the convolution neural network extraction and evaluation of text features mainly includes the following steps.

#### 3.1.1. Data Preprocessing

Through data preprocessing, the useless comments of English flipped classroom teaching quality evaluation text are filtered, and the filtered evaluation text is divided into short sentences. Through data filtering, we can avoid noise interference in the process of evaluating the quality of English flipped classroom teaching. When using convolution neural network to classify text evaluation, it is necessary to deal with comments composed of many short essays.

#### 3.1.2. Word Vector Training

Quantitatively transform the evaluation text, use the distribution formula word vector to represent the evaluation text, select a large-scale unsupervised method to train the distribution formula word vector so that the text that completes the quantitative transformation can reflect more grammatical information and semantics [19], and use the filtered evaluation text to realize the word vector training.

#### 3.1.3. Model Training and Testing

Extract part of the data and set it as the training set to complete the classification training. Mark the added labels through the model, compare the labels marked by the model with the existing labels, and adjust the model parameters through the error of the comparison results.

#### 3.1.4. Feature Extraction

The evaluation text represented by the word vector is input into the convolutional neural network, and the convolutional neural network outputs the corresponding label [20, 21]. Annotate the part of speech of the evaluation text, extract the nouns in the evaluation text, and obtain the feature word set. The tags forming the feature words are the same as those in the evaluation text.

### 3.2. Big Data Mining Technology

The same evaluation text contains multiple characteristic words, and the final evaluation index of English flipped classroom teaching quality needs to be determined by cluster analysis. Big data mining technology is to mine association rules with minimum confidence and minimum support in massive data [22]. Big data mining technology mining association rules mainly includes two parts: (1) mining frequent itemsets with minimum support in transaction database; (2) mining frequent itemsets used to generate association rules for English flipped classroom teaching quality evaluation. The Apriori algorithm is an important algorithm for mining frequent itemsets in data mining algorithms. This algorithm is used to obtain itemsets with support higher than the minimum support. The Apriori algorithm obtains *K* + 1 itemsets by using *K* itemsets through a layer-by-layer search method.

*B* represents the transaction database, *I*={*I*_1_, *I*_2_,…, *I*_*m*_} represents the itemset in the database, and *I*_*i*_ represents the elements in the itemset. *W*={*T*_1_, *T*_2_,…, *T*_*n*_} and *T*_*i*_, respectively, represent the transaction set and the elements contained therein and satisfy *T*_*i*_⊆*T*. Identify all transactions *T* with separate labels. The length or dimension of itemset in massive data indicates the number of elements contained in the itemset. When the number of elements in the itemset is K, it indicates that the itemset is a K-itemset. Set up a random English flipped classroom teaching quality transaction database *B* and mine its frequent itemsets. The process is as follows:

1. Calculate all 1 itemsets, represented by *C*_1_, and search all common 1 itemsets greater than or equal to the set minimum support, represented by *L*_1_.
2. Use the common 1 itemset to obtain the candidate 2 itemsets, which are represented by *C*_2_. Search for all 2 itemsets greater than or equal to the set minimum support from the obtained 2 itemsets, which are represented by *L*_2_.
3. According to the above process, use the obtained common 2 itemsets to obtain the candidate 3 itemsets, which are represented by *C*_3_. Search for all three itemsets greater than or equal to the set minimum support from the obtained three itemsets, which are represented by *L*_3_.
4. Repeat the above process until higher dimensional frequent items cannot be obtained, and terminate the iteration.

It can be seen from the above process that the Apriori algorithm obtains the final frequent itemset through continuous iteration [23], and too many candidate sets are formed in the search process, which has high complexity and low operation efficiency. The Boolean matrix is introduced into the Apriori algorithm to make it suitable for massive big data mining. The massive database of big data is prone to excessive memory. The database needs to be segmented, and the segmented database will be scanned in segments. Suppose that there are *N* transaction databases that complete the segmentation, which are represented by {*B*_1_, *B*_2_,…, *B*_*N*_}. It can be seen that the number of Boolean matrices is *N*, which corresponds to the transaction database that completes the segmentation one by one. The process of obtaining frequent itemsets using the Apriori algorithm optimized by the Boolean matrix is as follows:

(1) Set the number of copies of massive transaction database and determine the size of different copies. Initialize the loop variable to 1 and set the minimum support of the Apriori algorithm.
(2) Read *B*_*i*_ in the transaction database and map it to the Boolean matrix *R*_*i*_.
(3) Calculate the local minimum support of *R*_*i*_ for *B*_*i*_ using formula (1):
  (1) $$\begin{array}{c} \text{min}s_{i}=\text{min}\frac{B_{i}}{\left|B\right|}. \end{array}$$
In formula (1), |*B*_*i*_| and |*B*| represent the number of elements in the transaction database and the number of elements in the massive transaction database, respectively.
Obtain the corresponding row vector of the frequent itemset in *B*_*i*_ in the Boolean matrix *R*_*i*_ through the above formula, save the row vector obtained by searching, release the memory space of the Boolean matrix *R*_*i*_ update dataset, and obtain the updated matrix *R*_*i*_.
(4) Set *i*=*i*+1, and when the *i* ≤ *N* condition is met, return to step (2) to repeat the iterative calculation. Otherwise, go to step (5).
(5) Recombine the Boolean matrix *R*_*i*_ corresponding to all frequent itemsets in the transaction dataset *B*_*i*_, and establish a new Boolean matrix, which is represented by *R*=(*R*_1_, *R*_2_, ⋯,*R*_*N*_)^*T*^. Thirdly, search the minimum support of the new Boolean matrix, determine the corresponding row vector of the frequent itemset of the massive transaction database *B*, and obtain the frequent itemset that can finally evaluate the quality of English flipping classroom teaching.

### 3.3. Realize the Evaluation of English Flipped Classroom Teaching Quality

There are many commonly used algorithms for data mining, such as genetic algorithm, clustering algorithm, and Apriori algorithm [24]. Combined with the characteristics of English flipped classroom teaching quality evaluation, this paper uses the ID3 decision tree method to complete the data mining of teaching evaluation.

Decision trees can classify a large amount of data purposefully so as to obtain valuable potential information. The decision tree uses the sample attribute as the node, and the attribute value is the branch tree structure. The root node is the attribute with the largest amount of information in all samples, the middle node is the attribute with the largest amount of information in the root node, and the leaf node is the sample category value. In the ID3 algorithm [25], the entropy concept in information theory is applied to complete the selection of node attributes, and the decision tree is constructed through the attribute with maximum information gain, which ensures that the decision tree has the minimum number of branches and minimum redundancy.

The information entropy can be expressed as formula (2):

(2) $$\begin{array}{c} I\left(m\right)=\;\log_{2}\;p\left(m_{i}\right). \end{array}$$

In formula (2), *p*(*m*_*i*_) represents the proportion of samples with category *m*_*i*_ in the total samples. The information in the decision tree is binary coded [26], so the logarithm function is based on 2.

The conditional entropy of attribute *A* can be calculated by formula (3).

(3) $$\begin{array}{c} E\left(A\right)=p\left(m_{i}|v_{j}\right){\text{log}}_{2}\left(\frac{1}{I\left(m\right)}\right). \end{array}$$

In formula (3), *p*(*m*_*i*_*|v*_*j*_) is the conditional probability of category *m*_*i*_ when the value of attribute *A* is *v*_*j*_. The information gain of attribute *A* is calculated by formula (4):

(4) $$\begin{array}{c} Gain\left(A\right)=I\left(m\right)E\left(A\right). \end{array}$$

In formula (4), Gain(*A*) represents the coding information obtained from the branch of attribute *A*; that is, the value of *A* causes the expected compression of information entropy.

To sum up, the ID3 algorithm realizes attribute selection through information gain. Therefore, the use of gain rate can be expressed as formula (5):

(5) $$\begin{array}{c} Gain\;Ratio\left(A\right)=\frac{Gain\left(A\right)}{Split\;IitInfo\left(A\right)}. \end{array}$$

Suppose *D* represents the training sample set, *A* represents an attribute in *D*, *A* contains *n* noncoincident values {*v*_1_, *v*_2_,…, *v*_*n*_}, and *D* is divided into *n* subsets *D*_1_, *D*_2_,…, *D*_*n*_; then, the value of the training sample in *D*_*i*_ in *A* can be expressed as *v*_1_. The process of decision tree mining is to automatically construct a decision tree from the data of the training set through the above method and then judge any instance according to the obtained decision tree.

Taking the six evaluation contents in Table 1 as the sample data of evaluation indicators and training, the number of teachers participating in the evaluation is 1∼6 to evaluate the quality of English flipped classroom teaching, and the results are divided into five grades as follows:

1. A: excellent, with a score of 90–100.
2. B: good, with a score of 80–89.
3. C: medium, with a score of 70–79.
4. D: pass, with a score of 60–69.
5. E: fail, score below 60.

The evaluation results of teachers are shown in Table 2.

It can be seen from Table 2 that the information entropy required by the sample can be expressed as formula (6):

(6) $$\begin{array}{c} I\left(S\right)=\frac{1}{6}{\text{log}}_{2}\left(\frac{1}{6}\right). \end{array}$$

The information entropy of each attribute can be obtained through formula (6). The attribute with the largest information gain is taken as the node test attribute. The branch of the corresponding attribute value is constructed downward from the root node, and the ID3 decision tree algorithm is continued to be used for further division. If the path from the root node to the current node contains all attributes, the algorithm ends, and the whole process of comprehensive evaluation is completed.

## 4. Experimental Test

In order to verify the effectiveness of the designed English flipped classroom teaching quality evaluation model based on big data analysis, a teacher's English flipped classroom teaching course is selected as the test object, and the main data comes from the intelligent English flipped classroom teaching system. A total of 8795 people attended the English flipped classroom teaching. After the students completed the class, they did feedback on the evaluation text, deleted the useless evaluation text and garbage evaluation text, and collected a total of 8564 effective evaluation texts. The experimental tests were carried out by using the methods of papers [6, 7].

### 4.1. Model Inspection

The grid search method is selected to determine the hyperparameters. The results of the determined convolution neural network hyperparameters for extracting and evaluating text features are shown in Table 3.

**Figure 5:**
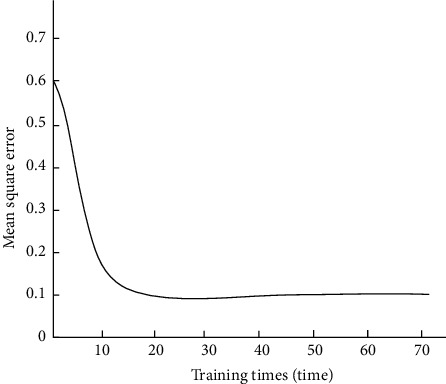
Convergence results of convolutional neural network.

According to the parameters determined in Table 3, the convolutional neural network is trained and tested by the tenfold cross-validation method. The convergence of the convolutional neural network is shown in Figure 5.

As can be seen from the experimental results in Figure 5, the convolutional neural network is used to extract the features contained in the evaluation text in this method, and the rapid convergence can be achieved only after about 15 times of network training. It shows that the convolutional neural network selected in this method has a high convergence speed and can improve the operational performance of the English flipped classroom teaching quality evaluation model.

### 4.2. Algorithm Performance Test

In order to make the comparative data more accurate, the comprehensive evaluation value given by several teaching supervision experts in many lectures is used as the verification basis of the accuracy of this method. The comparison data between the evaluation results obtained by using this method and the comprehensive evaluation results of several expert groups are shown in Table 4.

From the data in Table 4, it can be concluded that the evaluation results given by this method are close to those of the expert group. Therefore, it can be seen that the system is a feasible and reasonable teaching quality evaluation system.

The expert evaluation method is used as the standard to evaluate the quality of English flipped classroom teaching. This method is used to evaluate the evaluation accuracy and recall rate of English flipped classroom teaching quality in different evaluation text sizes. In order to intuitively show the evaluation effect of this method, this method is compared with the models in [6] and [7]. The comparison results are shown in Table 5.

From the data in Table 5, it can be concluded that using this method to evaluate the quality of English flipped classroom teaching has high evaluation accuracy and recall rate for different evaluation text sizes. Accuracy and recall rate are important evaluation indexes to evaluate the performance of machine learning methods. The evaluation accuracy and recall rate of this method are higher than 98%, which can meet the needs of big data English flipping classroom teaching quality evaluation. The evaluation accuracy and recall rate of the other two models decrease with the increase of the number of evaluation texts, indicating that the other two methods cannot adapt to massive data processing, and the operation level decreases when the amount of data increases.

### 4.3. Actual Verification

In the evaluation, the designed model text scoring standard is adopted, and the full score is 25 points. One class is selected to apply the teaching mode of this paper, and the remaining two classes are selected to adopt the traditional teaching mode. The average composition score data of the three classes are shown in Table 6.

According to the average composition score data of the three classes in Table 6, the average composition score of the experimental class is significantly higher, the average composition score of the control class 2 is in the middle, and the average composition score of the control class 1 is the lowest. It shows that the designed flipped classroom teaching mode of English writing is the most effective and can improve students' composition scores.

**Figure 6:**
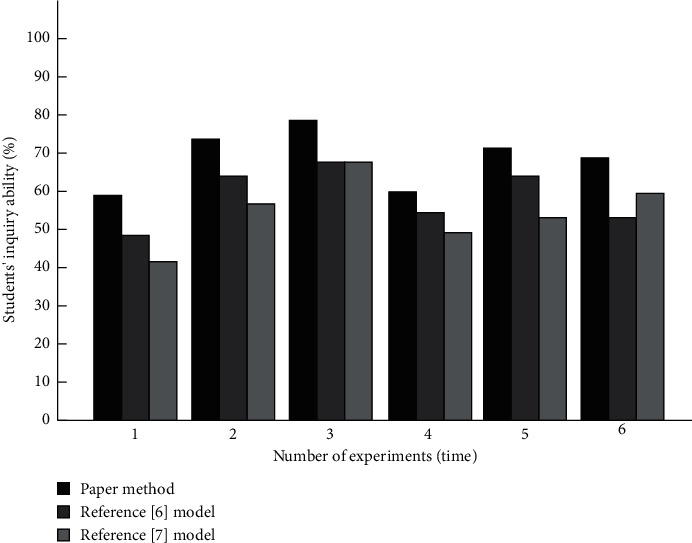
Comparison of students' inquiry ability.

The comparison results of students' inquiry ability after learning the three methods are shown in Figure 6.

It can be seen from Figure 6 that the students' inquiry ability improved more after applying the design teaching mode, which is better than that of the students applying the comparative method.

**Figure 7:**
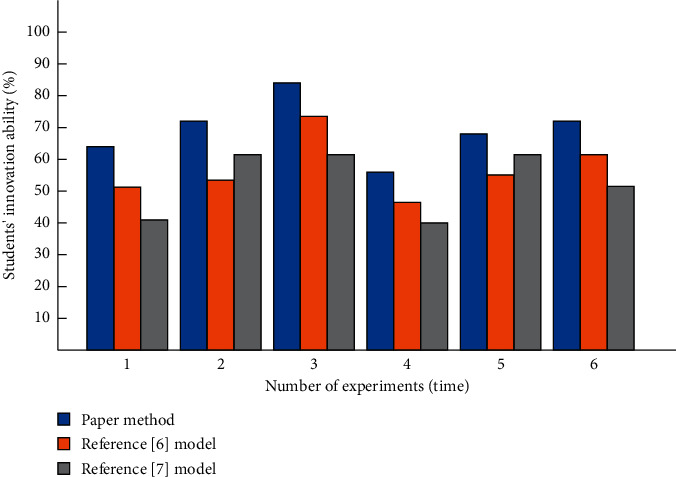
Comparison of students' innovation ability.

The comparison results of students' innovation ability with the three methods are shown in Figure 7.

It can be seen from the analysis of Figure 7 that the innovation ability of students applying the design teaching mode is much higher than that in the past because, under the design teaching mode, students can experiment by themselves and strengthen their communication with teachers, so as to improve students' innovation ability. After the application of comparative teaching mode, although the students' innovation ability has improved, the improvement in innovation ability is not high.

**Figure 8:**
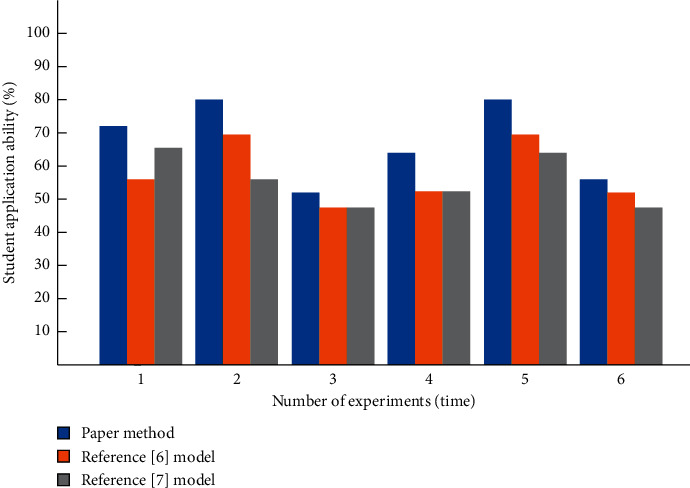
Comparison of students' application ability.

The comparison results of students' application ability after applying the three methods are shown in Figure 8.

It can be seen from Figure 8 that the application ability of students after applying the traditional two systems is improved less, while the application ability of students after applying the system designed this time is improved higher, which shows that students have accumulated small phenomena related to English in life and can connect their learned knowledge with practice.

**Figure 9:**
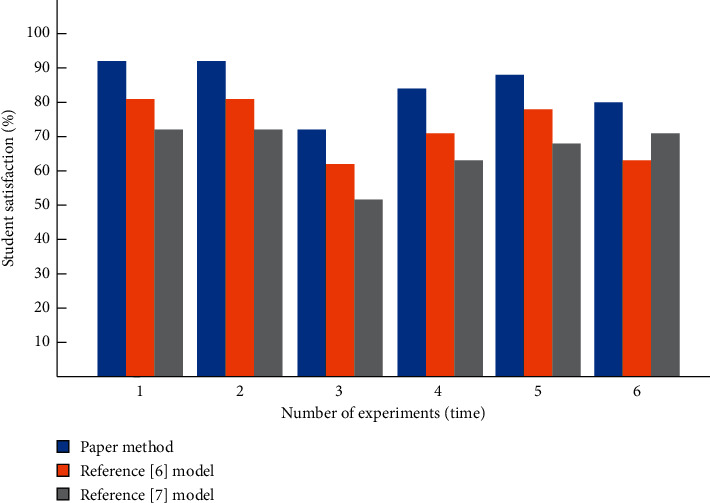
Comparison of students' satisfaction with teaching mode.

The comparison results of students' satisfaction after applying the three methods are shown in Figure 9.

As can be seen from Figure 9, students' satisfaction is greatly improved after applying the designed English flipped classroom teaching mode because, in the flipped classroom teaching mode, students become the leaders in the classroom and stimulate students' curiosity. Although the satisfaction of students applying the comparative method has improved, it is lower than that of students applying the teaching mode designed this time.

Therefore, the above experiments can prove that the designed English flipped classroom teaching model can improve students' inquiry ability, innovation ability, and application ability, and students are more satisfied with the teaching model.

## 5. Conclusion

English flipped classroom teaching quality has high subjectivity, which is difficult to accurately quantify as a qualitative problem. The big data mining method is used to mine the massive data related to English flipped classroom teaching, determine the evaluation index of English flipped classroom teaching quality, and obtain the quantitative scoring results by using the determined evaluation index of English flipped classroom teaching quality. The use of big data technology provides theoretical support for the accurate evaluation of English flipped classroom teaching quality. Through the experimental verification, the evaluation accuracy and recall rate of the evaluation method studied are higher than 98%, which can realize the accurate evaluation of English flipped classroom teaching quality.

## Figures and Tables

**Table 1:** Evaluation criteria for teachers' learning tasks.

Serial number	Project	Evaluation criteria	Weight (%)
1	Achieve the goal (Q1)	Internalizing knowledge and expanding ability (Q11)	10
2	Learning methods (Q2)	Ability to help students complete learning tasks (Q21)	10
3	Preview of classroom learning formula (Q3)	It is conducive to arousing students' enthusiasm to complete preclass tasks seriously (Q31)	10
4	Learning tasks (Q4)	Whether advanced homework is conducive to students' internalization of knowledge (Q41)	20
		Based on the cognitive level reached by “advanced,” the difficulty is greater than that of advanced homework (Q42)	40
		Collaborative inquiry comes from real situations (Q43)	10
Total score		Comprehensive score	

**Table 2:** Teacher evaluation results.

Number	Q11	Q21	Q31	Q41	Q42	Q43	Total
1	B	B	A	B	A	B	Good
2	B	A	B	B	B	A	Good
3	B	B	B	B	B	B	Good
4	C	B	C	C	C	C	Secondary
5	B	C	C	C	B	B	Secondary
6	A	A	A	B	A	A	Excellent

**Table 3:** Superparameter setting.

Superparametric index	Numerical results
Number of feature maps	100
L2 regularization coefficient	4
Convolution kernel size	(6,350), (7,350), (8,350)
Dropout scale	0.7
Batch value	9

**Table 4:** Comparison of evaluation results.

Sample number	Expert evaluation value	Evaluation value of this method	Expert evaluation results	The evaluation results of this method
1	62.88	62.79	Pass	Pass
2	72.34	72.46	Secondary	Secondary
3	84.57	84.57	Good	Good
4	93.67	94.11	Excellent	Excellent
5	77.31	77.31	Secondary	Secondary

**Table 5:** Comparison of evaluation results of different evaluation text sizes.

Evaluation text size (MB)	Paper method (%)		Reference [6] model (%)		Reference [7] model (%)	
	Accuracy	Recall	Accuracy	Recall	Accuracy	Recall
100	98.46	99.36	96.63	95.53	96.96	95.95
200	99.36	99.85	94.63	94.73	95.75	94.37
300	99.55	98.56	92.76	93.96	94.63	93.96
400	98.36	98.63	93.63	92.96	93.63	92.95
500	98.48	98.75	91.63	91.63	92.27	91.65
600	98.95	98.27	94.63	89.96	91.53	90.69
700	99.07	98.54	90.36	88.57	90.68	88.57
800	99.86	98.69	91.63	87.63	88.65	87.63
900	99.55	98.84	89.05	87.05	87.75	85.75
1000	98.76	98.45	88.75	85.75	86.63	84.72

**Table 6:** Average composition score data of three classes.

Composition	Average composition score		
	Experimental class	Control class 1	Control class 2
One	22	15	18
Two	23	16	17
Three	24	14	19
Four	22	15	18
Five	22	14	17

## Data Availability

The raw data supporting the conclusions of this paper can be obtained from the corresponding author without undue reservation.
